# Correction: Evolution of our understanding of cell volume regulation by the pump-leak mechanism

**DOI:** 10.1085/jgp.20181227402282019c

**Published:** 2019-03-06

**Authors:** Alan R. Kay, Mordecai P. Blaustein

Vol. 151, No. 4, April 1, 2019. 10.1085/jgp.201812274.

After publication, we discovered that there was an error in the units of the pump rate (*p*) in the legend to Figure 2 and in the x-axis label in Figure 3. The authors would like to thank Dr. Stephen Baylor for pointing out the error.

**Figure 2.**
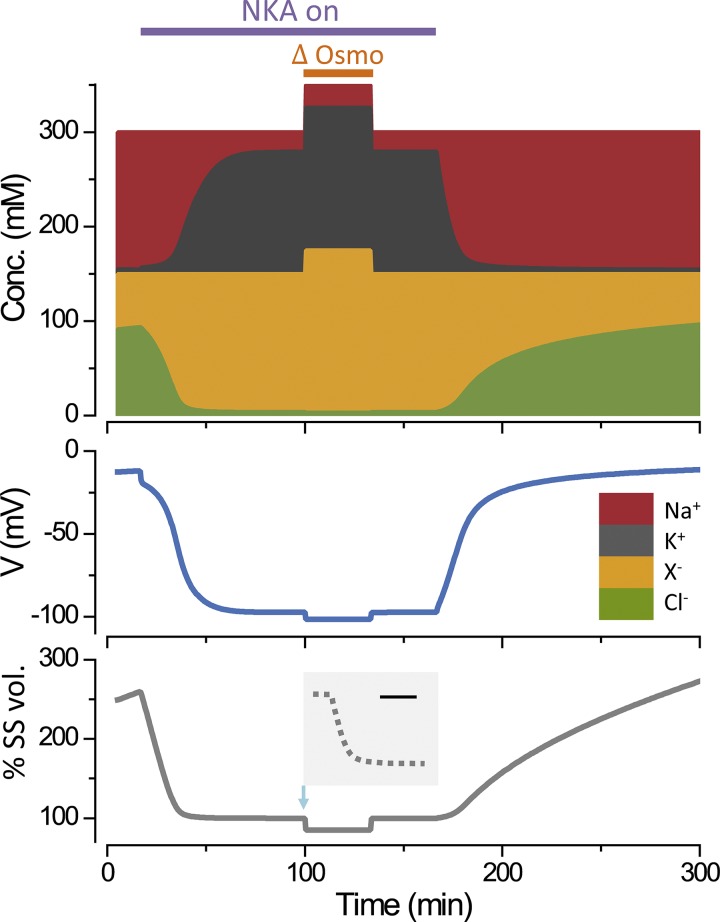
The action of the NKA stabilizes cell volume. The line labeled “NKA on” indicates when the pump is turned on and then off (*p* = 0.5 µC cm^−2^ s^−1^). The composition of the extracellular solution is changed during the period indicated by “ΔOsmo,” to 350 from 300 mOsm/liter, by adding an impermeant uncharged molecule. The intracellular ion concentrations ([Conc.], top panel) are plotted as a function of time, with the concentrations stacked on top of one another. The voltage (middle panel) and cell volume (lower panel) are plotted on the same timescale. The inset on the lower panel shows the change in volume, at a higher temporal resolution (bar, 0.5 s), when the osmolarity is increased. When the pump is turned off, the cell volume increases, slowly but without limit, showing that the system is unstable. The volume is expressed as a percentage of the steady-state volume (% SS vol.). The equations and parameters are given in the Appendix.

**Figure 3.**
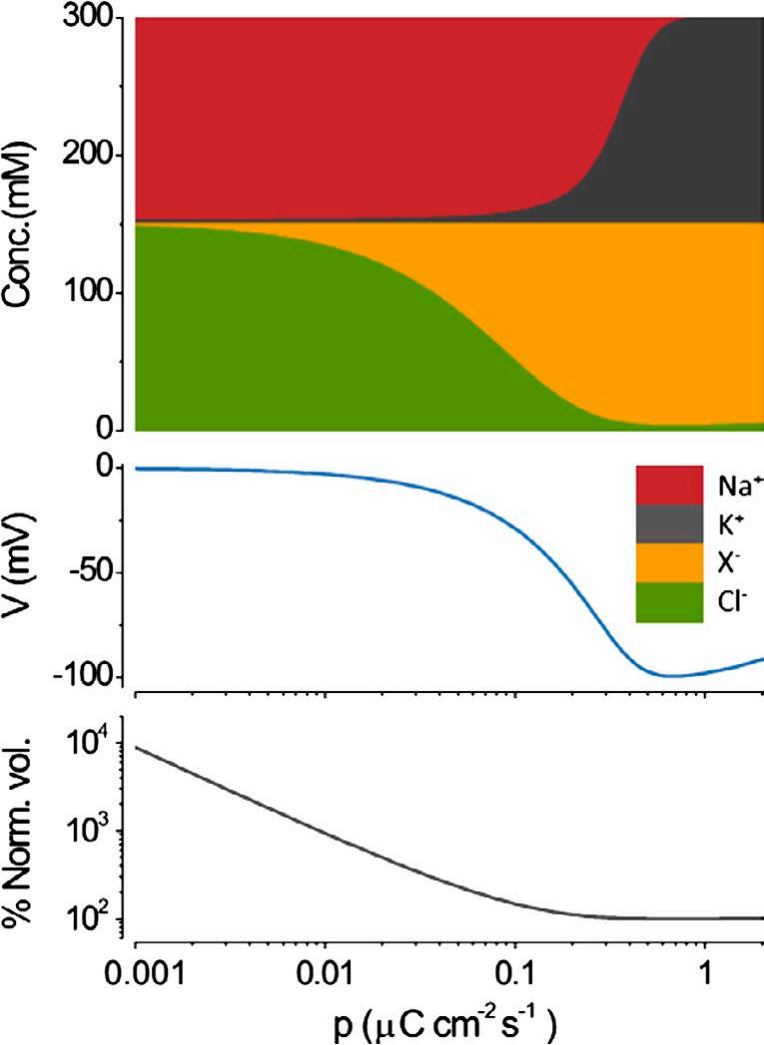
Constraints on the PLM. The steady-state intracellular ion concentrations ([Conc.], top), voltage (middle), and volume (bottom) are plotted as a function of the NKA pump rate (*p*). The concentrations are stacked as in Fig. 2. The steady-state solutions shown are the analytical Keener–Sneyd solution given in the Appendix. The hyperpolarization induced by the action of the NKA drives Cl^−^ out of the cell. Water moves out of the cell, preserving osmotic and charge balance, leading to a decrease in volume and an increase in the concentration of *X*^*z*^. As *p* is increased further, K^+^ progressively substitutes for Na^+^. Osmotic balance is shown again by the fact that the sum of all intracellular ions at any *p* equals the extracellular osmotic strength. Electroneutrality is also preserved at all *p*; the sum of anions = sum of cations; *z* = −1. The volume is normalized by that at *p* = 2 (% Norm. vol.). The figure has been modified from Kay (2017), with a temperature of 37°C rather than 25°C.

Both the HTML and PDF versions of the article have been corrected. These errors appear only in PDF versions downloaded on or before March 6, 2019.

